# Multicenter Study of Brucellosis in Egypt 

**DOI:** 10.3201/eid1412.071452

**Published:** 2008-12

**Authors:** Hassan Samaha, Meshref Al-Rowaily, Ramadan M. Khoudair, Hossam M. Ashour

**Affiliations:** Aljouf University, Aljouf, Saudi Arabia (H. Samaha, M. Al-Rowaily); Animal Health Research Institute, Cairo, Egypt (R.M. Khoudair); Cairo University, Cairo (H.M. Ashour)

**Keywords:** Brucella, serodiagnosis, zoonosis, prevalence, Rose Bengal test, Egypt, dispatch

## Abstract

Brucellosis causes appreciable economic losses in livestock. Examination of milk and tissues from animals in Egypt for *Brucella* spp. showed increased prevalence rates of serologically reactive animals. All isolates were *B. melitensis* biovar 3. One *Brucella* sp. was isolated from milk of serologically nonreactive buffaloes.

Brucellosis is one of the major zoonotic infections worldwide (*1*). It is caused by gram-negative coccobacilli of the genus *Brucella* and affects cattle, sheep, goats, and other livestock (*2*,*3*). Since the discovery of *Brucella melitensis* by David Bruce in 1887, several species have been identified, such as *B*. *abortus* (which infects cattle), *B*. *melitensis* (which infects sheep and goats), *B*. *suis*, *B*. *neotomae*, *B*, *ovis*, and *B*. *canis* (*2*,*4*). Although brucellosis has been controlled in most industrialized countries, it remains a major problem in the Mediterranean region, western Asia, Africa, and Latin America (*1*). It can cause appreciable economic losses in the livestock industry because of abortions, decreased milk production, sterility, and veterinary care and treatment costs (*2*).

Brucellosis was first reported in Egypt in 1939 (*5*). Control programs for brucellosis in Egypt have used 2 methods: vaccination of all animals and slaughter of infected animals with positive serologic results. The difficulty of accurately detecting all infected animals, especially carriers, is a major limitation of these programs. To enhance efficiency of brucellosis-specific prophylaxis, early detection of brucellosis by highly sensitive and specific methods is needed.

Egypt has mixed populations of sheep, goats, cattle, and buffaloes. The number of buffaloes in Egypt is higher than in any other country in the Near East region (*5*). In addition to high prevalence rates of *B*. *melitensis* infections in sheep and goats, *B*. *melitensis* infections of cattle and buffaloes have increased in Egypt (*5*). Our investigation sought to determine the epidemiology of brucellosis in several governorates in Egypt by using different serologic tests, as well as bacteriologic tests, to identify *Brucella* spp. organisms isolated from milk and tissue specimens of sheep, cattle, goats, and buffaloes.

## The Study

We studied 4,482 animals (1,966 cattle, 1,237 buffaloes, 813 sheep, and 366 goats) from production and breeding farms in various governorates in Egypt during 2007; the animals had no history of having been tested for brucellosis. Milk and tissue samples obtained from all animals were examined for *Brucella* spp. We used serologic tests recommended by the National Brucella Committee, which represents the general organization of veterinary services, veterinary laboratories, and universities in Egypt (*5*). The buffered acidified plate antigen (BAPA) test, the Rose Bengal plate test, the standard tube agglutination test, and the Rivanol test were used as described (*6*–*8*).

Direct culture of milk under aseptic conditions was conducted as follows: ≈20 mL of milk was centrifuged at 1,620 × *g* for 10 min, and the sediment cream mixture was placed on *Brucella* spp. agar plates containing an antimicrobial drug supplement. Tissue specimens obtained from internal organs, supramammary lymph nodes, and udders were cultured in the same media and incubated at 37°C in an atmosphere of 10% CO_2_. Cultured plates were examined for *Brucella* spp. growth on day 4 and daily for 4 weeks. Suspected colonies were further identified and subcultured on *Brucella* spp. agar slants. We identified *Brucella* spp. isolates according to morphologic characteristics, microscopic appearance, and reactions with positive sera. *Brucella* spp. isolates were typed according to their CO_2_ requirement, H_2_S production, growth in the presence of dyes, reaction with monospecific sera (immunoglobulin [Ig] A and IgM), and bacteriophage typing (Tiblisi phage; Central Veterinary Laboratory, Wybridge, UK) as described (*7*).

Results obtained for different animal groups are shown in Table 1. Prevalence of brucellosis in cattle was 5.44% by the BAPA test; highest prevalence was in Benisuef (7.77%) and Monofia (7.14%). Prevalence of brucellosis in buffaloes was 4.11% by the BAPA test; highest prevalence was in Benisuef (6.93%) and Qalioubia (5.34%). Prevalence of brucellosis in sheep was 5.41% by the BAPA test; highest prevalence was in Benisuef (6.91%) and Giza (5.81%). Prevalence of brucellosis in goats was 3.55% by the BAPA test; highest prevalence was in Monofia (6.35%) and Benisuef (5.75%).

**Table 1:** Serodiagnostic test results for brucellosis in animals, Egypt, 2007*

Serum source	Location	No. tested	Serologic test, no. positive (%)			
			BAPA	RBP	SA	Rivanol
Cattle	Alexandria	333	17 (5.11)	15 (4.5)	13 (3.9)	13 (3.9)
	Behera	374	11 (2.94)	11 (2.94)	10 (2.67)	9 (2.41)
	Monofia	280	20 (7.14)	18 (6.43)	17 (6.07)	15 (5.36)
	Qalioubia	221	14 (6.33)	12 (5.43)	12 (5.43)	11 (4.98)
	Giza	346	15 (4.34)	15 (4.34)	14 (4.05)	14 (4.05)
	Benisuef	309	24 (7.77)	22 (7.12)	21 (6.8)	21 (6.8)
	Assiut	103	6 (5.83)	5 (4.85)	6 (5.83)	5 (4.85)
	Total	1,966	107 (5.44)	98 (4.98)	93 (4.73)	88 (4.48)
Buffaloes	Alexandria	137	6 (4.38)	6 (4.38)	6 (4.38)	6 (4.38)
	Behera	397	7 (1.76)	5 (1.26)	5 (1.26)	5 (1.26)
	Monofia	210	10 (4.76)	8 (3.81)	6 (2.86)	7 (3.33)
	Qalioubia	131	7 (5.34)	6 (4.58)	7 (5.34)	6 (4.58)
	Giza	198	8 (4.04)	8 (4.04)	8 (4.04)	7 (3.54)
	Benisuef	231	16 (6.93)	14 (6.06)	14 (6.06)	14 (6.06)
	Assiut	33	1 (3.03)	0	0	0
	Total	1,337	55 (4.11)	47 (3.52)	46 (3.44)	45 (3.37)
Sheep	Behera	210	11 (5.24)	10 (4.76)	10 (4.76)	10 (4.76)
	Monofia	81	2 (2.47)	0	0	0
	Qalioubia	133	6 (4.51)	6 (4.51)	6 (4.51)	6 (4.51)
	Giza	172	10 (5.81)	9 (5.23)	9 (5.23)	9 (5.23)
	Benisuef	217	15 (6.91)	14 (6.45)	14 (6.45)	14 (6.45)
	Total	813	44 (5.41)	39 (4.8)	39 (4.8)	39 (4.8)
Goats	Behera	55	1 (1.82)	0	0	0
	Monofia	63	4 (6.35)	2 (3.17)	2 (3.17)	2 (3.17)
	Qalioubia	103	3 (2.91)	2 (1.94)	2 (1.94)	2 (1.94)
	Giza	58	0	0	0	0
	Benisuef	87	5 (5.75)	4 (4.6)	4 (4.60)	4(4.6)
	Total	366	13 (3.55)	8 (2.19)	8 (2.19)	8(2.19)

*BAPA, buffer acidified plate antigen; RBP, Rose Bengal plate; SA, standard tube agglutination.

Prevalence of a serologic reaction was 4.98% for cattle, 3.52% for buffaloes, 4.8% for sheep, and 2.19% for goats by the Rose Bengal plate test. Prevalence of a serologic reaction was 4.73% for cattle, 3.44% for buffaloes, 4.8% for sheep, and 2.19% for goats by the standard tube agglutination test. Prevalence of a serologic reaction was 4.48% for cattle, 3.37% for buffaloes, 4.8% for sheep, and 2.19% for goats by the Rivanol test. The highest prevalence for cattle, buffaloes, sheep, and goats by any of the 4 tests was in Benisuef, except for the BAPA test in goats, which showed highest prevalence rates in Monofia.

Isolation of the causative agent is still the standard diagnostic method for brucellosis (*9*). Thus, for definitive and confirmative diagnosis of serologically reactive animals, bacteriologic isolation and identification of *Brucella* spp. were performed. Results of bacteriologic isolation from milk and tissues all animals are shown in Table 2. A total of 47 isolates of *Brucella* spp. were identified; all isolates were *B*. *melitensis* biovar 3. Isolation of *Brucella* spp. confirmed active brucellosis in the animals tested. A *Brucella* spp. was also isolated from milk samples from serologically nonreactive buffaloes in Benisuef.

**Table 2:** Prevalence of *Brucella* spp. in milk or tissues of animals, Egypt, 2007*

Location	Cattle, no. positive/no. tested						Buffaloes, no. positive/no. tested						Sheep, no. positive/no. tested			Goats, no. positive/no. tested	
	Milk			Tissue			Milk			Tissue			Tissue			Tissue	
	SRA	SNRA		SRA	SNRA		SRA	SNRA		SRA	SNRA		SRA	SNRA		SRA	SNRA
Alexandria	2/10	0/11		1/5	0/5		1/6	0/19		1/5	0/5		0	0		0	0
Behera	2/9	0/9		1/5	0/5		1/5	0/20		1/5	0/5		1/5	0/5		0	0/5
Monofia	4/20	0/12		1/5	0/5		1/7	0/18		2/5	0/5		0	0/5		0/2	0/5
Qalioubia	2/20	0/10		0/5	0/5		2/6	0/3		1/5	0/5		1/5	0/5		1/2	0/5
Giza	4/20	0/10		0/5	0/5		1/7	0/6		1/5	0/5		1/5	0/5		0	0
Benisuef	6/20	0/21		2/5	0/5		1/10	1/15		0/5	0/5		1/5	0/5		1/4	0/5
Assiut	1/5	0/7		1/5	0/5		0	0/5		0	0		0	0		0	0
Total	21/104	0/80		6/35	0/35		7/41	1/86		6/30	0/30		4/20	0/25		2/8	0/20

*SRA, samples from serologically reactive animals; SNRA, samples from serologically nonreactive animals.

## Conclusions

We observed an increase in animals serologically reactive for *Brucella* spp. in Egypt in 2007 (Table 1). Prevalence rates in cattle, buffaloes, sheep, and goats were generally higher in Benisuef than in other governorates. Variations in infection in different governorates may be attributed to environmental factors and stress, which may modulate susceptibility to infection.

Increased prevalence of brucellosis in cattle and buffaloes in Egypt can be attributed to raising sheep and goats with cattle or buffaloes in villages. Most sheep or goat flocks in Egypt are mobile. Movement of infected sheep or goats can contaminate pastures and spread brucellosis to other animals (e.g., cattle or buffaloes) in other herds or areas. This movement is a major risk factor for failure of brucellosis eradication programs. Elimination or control of infection in sheep and goat flocks can reduce spread of the disease in cattle and buffaloes.

All *Brucella* isolates were *B*. *melitensis* biovar 3. This finding is consistent with reports of *B*. *melitensis*, particularly biovar 3, being the main cause of brucellosis in animals and humans in many countries (*5*). Isolation and identification of 1 *Brucella* spp. from milk samples of serologically nonreactive buffaloes in Benisuef emphasize the need to routinely check milk samples. Some microorganisms, which can escape identification by not causing appreciable serologic responses, can localize in the udder and be isolated from milk samples.

We recently reported prevalence of human brucellosis in Egypt as high as 8% in high-risk populations (*10*). Our findings emphasize the need for continuous national surveillance programs for control and prevention of brucellosis in Egypt and other affected countries. Measures should be established to control spread of brucellosis, especially in mobile flocks. These measures should include identification of infected animals by periodic examination of flocks or newly purchased animals, application of testing and slaughter policies, adoption of vaccination programs, and strict quarantine measures. Sheep farmers should also be notified about transmission of brucellosis from sheep to cattle and buffaloes. Educational programs about brucellosis are important for livestock owners and consumers.
